# Critical assessment of on-premise approaches to scalable genome analysis

**DOI:** 10.1186/s12859-023-05470-2

**Published:** 2023-09-21

**Authors:** Amira Al-Aamri, Syafiq Kamarul Azman, Gihan Daw Elbait, Habiba Alsafar, Andreas Henschel

**Affiliations:** 1 Department of Electrical Engineering and Computer Science, College of Engineering, Khalifa University, P.O. Box 127788, Abu Dhabi, United Arab Emirates; 2 Department of Biology, College of Arts and Sciences, Khalifa University, P.O. Box 127788, Abu Dhabi, United Arab Emirates; 3 Center for Biotechnology (BTC), Khalifa University, P.O. Box 127788, Abu Dhabi, United Arab Emirates; 4 Department of Biomedical Engineering, Khalifa University, P.O. Box 127788, Abu Dhabi, United Arab Emirates

**Keywords:** Genomic data science, Big data, Genomic databases, SQL, VCF, NoSQL, Horizontal scaling

## Abstract

**Background:**

Plummeting DNA sequencing cost in recent years has enabled genome sequencing projects to scale up by several orders of magnitude, which is transforming genomics into a highly data-intensive field of research. This development provides the much needed statistical power required for genotype–phenotype predictions in complex diseases.

**Methods:**

In order to efficiently leverage the wealth of information, we here assessed several genomic data science tools. The rationale to focus on on-premise installations is to cope with situations where data confidentiality and compliance regulations etc. rule out cloud based solutions. We established a comprehensive qualitative and quantitative comparison between BCFtools, SnpSift, Hail, GEMINI, and OpenCGA. The tools were compared in terms of data storage technology, query speed, scalability, annotation, data manipulation, visualization, data output representation, and availability.

**Results:**

Tools that leverage sophisticated data structures are noted as the most suitable for large-scale projects in varying degrees of scalability in comparison to flat-file manipulation (e.g., BCFtools, and SnpSift). Remarkably, for small to mid-size projects, even lightweight relational database.

**Conclusion:**

The assessment criteria provide insights into the typical questions posed in scalable genomics and serve as guidance for the development of scalable computational infrastructure in genomics.

## Introduction

The past few years have witnessed a rapid progression in the study and understanding of human genetic variations. This has resulted in an incredible wealth of information that expanded the knowledge of interpreting genetic variations and emphasizing their diversity. The anticipated growth of genomic data considering 100s of millions of genomes sequenced by 2025 is estimated to reach data volumes in the order of Exabytes [1]. Genetic variations may differ in their characteristics and the forms they take in the human genome [2]. These differences are the key factors to unravel underlying phenotypes and define disease susceptibility in different individuals [3–6]. However, as many phenotypes are complex and polygenic, statistical power analysis has shown that huge sample sizes are required [7]. For example, a genome wide association study for human height variation has recently been conducted and involved 5.4 million samples [8]. While the actual DNA sequencing (and genotyping array) technology seems to scale with this demand, it is not clear how downstream Genomics analysis can keep up and leverage Big Data technologies of recent decades in the best possible way. The problem is exacerbated by a community that has built its foundation in flat-file based ecosystems, at a time when data volumes were orders of magnitudes smaller. The variant call format (VCF) file is a popular flat-file format that holds genetic variation information in a tabular form. VCF files feature metadata columns providing detailed information pertaining to a variant and the set of samples carrying that variant. In studies revolving around a cohort, the VCF file links together the variant information and the genotypes of the individuals in the study.

The tabular paradigm of the VCF conforms well to non-technical researchers, as it imitates a spreadsheet environment. The highly-exploited INFO column, a loosely structured data field into which variant annotations are stored, is one of the creature comforts of the VCF. Many annotation tools implement syntactic conventions, however, they are not enforced by the VCF specifications and different tools follow conflicting conventions. As a result, processing and indexing all columns of VCF files and extracting the unstructured data into a digest becomes nontrivial, especially as the size of VCFs becomes a daunting task.

Many genomic tools provide variant analysis solutions using VCF files but lack functionality when it comes to managing the variant data, let alone multiple projects and heavy data querying [9].

Genomic data science algorithms and databases are co-evolving with the abundance of data engendered by next-generation sequencing (NGS) as a means of performing clinical studies. Bread-and-butter research like genome-wide association studies (GWAS) or personalized medicine relies on statistical measures that improve with larger cohorts. This is demonstrated in ongoing successes such as the 100,000 Genomes Project [10] or the BioBank Japan Project [11] but suffers from infrastructural challenges at scale. The 100,000 Genomes Project recently introduced a sparse relational database Rareservoir, which focuses on large amounts of rare variants only [12]. The authors argue for the use of relational databases, but avoid horizontal scaling by variant reduction assuming that causative, contributing variants are rare and known in advance. Big data solutions seemingly remedy these issues as evident in the growing adoption of distributed paradigms in recent releases of bioinformatics software (e.g., GATK [13], etc).

Presently, several studies have been steered towards presenting solutions for assessing and processing variants. There are different genomic data science tools that exist to manipulate and extract genomic information for interpreting the relevance of identified variants. These tools range from simple flat-file-based solutions e.g (BCFtools [14], SnpSift [15], VCFtools [16]), slivar [17] to database-enabled platforms e.g. (GEMINI [4], Hail [18], OpenCGA [19] and VCF-Miner [20]).

A clear dichotomy in the toolbox is that flat-file manipulation programs are favored over managed platforms, as evident in the prevalence of such tools in the literature.

Current solutions and processing pipelines are not all designed to work as standalone tools. This entails depending on external variant annotation sources and third-party annotation tools (e.g., ANNOVAR [21], VEP [22], VariantAnnotation [23]) to process a VCF file effectively. Among the current solutions and processing pipelines, several are structured for cloud-based models. While this is driven by the advantages of cloud infrastructure, on-premises deployments are desirable to comply with legal restrictions; for example, a country’s ban on the movement of human genetic sequences outside digital borders. This work focused on on-premise solutions to explore areas of deployments as it is understudied, to the best of our knowledge. Interested readers can find more on cloud-based genomics solutions in a comprehensive review by Shi and Wang [24].

Another crucial attribute of a VCF processing platform is to what extent it enables a user to express complex queries. Undoubtedly, the expressive power of queries is directly linked to the wealth of information available in the VCF file, especially in the INFO column where annotations reside. It is worth noting that many annotations utilize hierarchical structures such as ontologies (e.g., Gene Ontology [25], SNPEff mutation ontology [15]) and taxonomies (e.g., metabolic pathway hierarchies [26], ClinVar [27], ICD [28]). A modern query system should be capable of harvesting semantic annotations that are intrinsically taxonomy-oriented. For instance, selecting variants for *Parkinson’s disease* which is a member of *degenerative nervous system disorders*, which is, in return, under the umbrella of *neurological disorders*. Algorithmically, this can then be used to explore hierarchical feature spaces to perform genotype-phenotype predictions, as for the example done in [29].

In this work, we focused on evaluating and assessing the performance of different on-premise genomic data science approaches for human genomics. We bring a comprehensive comparison based on numerous feature categories such as storage, query speed, scalability, annotation, manipulating data (filtering, extracting), visualization method, data output representation, and the availability of these tools as open-source or peer-reviewed articles. Addressing the query speed is essential as it is not often assessed in current reviews of the literature [30, 31]. A common metric of these tools is the ability to load and process a whole parsed VCF file. We have omitted tools like VCF-Miner as it handles each chunk of the VCF file separately; hence it doesn’t allow for a fair comparison with other analysis tools. This work intends to bring a solid foundation for bioinformaticians and other researchers interested in genomics applications to identify ideal solutions that match their purposes.

## Methods

In this section, we address a common genomics data science workflow on which the tools are applied. We then explain the VCF file format and its contents. In particular, the INFO column of the VCF file format is elaborated as it is the most crucial part for variant annotation. We then introduce the five data science tools used for the comparison. Finally, we define each of the highlighted features and how each feature fits in the selected tools.

### General workflow

**Fig. 1:**
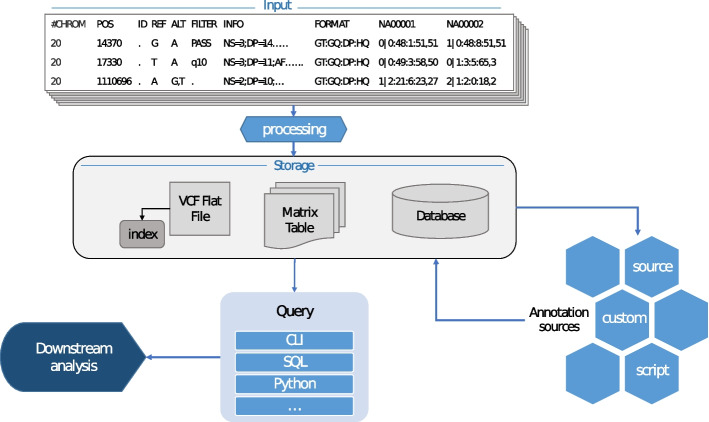
The general workflow of a genomics data science solution. The input is a VCF file after a variant calling pipeline which could undergo transformation into a storage system. Variants are then annotated with a variety of sources and fed back into the storage. The contents of the VCF file can be queried via a client or a program for later analysis

Existing data science genomics solutions follow a common workflow to some extent. The diagram in Fig. 1 shows the abstract illustration for the generalized approach followed by most VCF manipulation tools studied in this critical assessment.

**Fig. 2:**
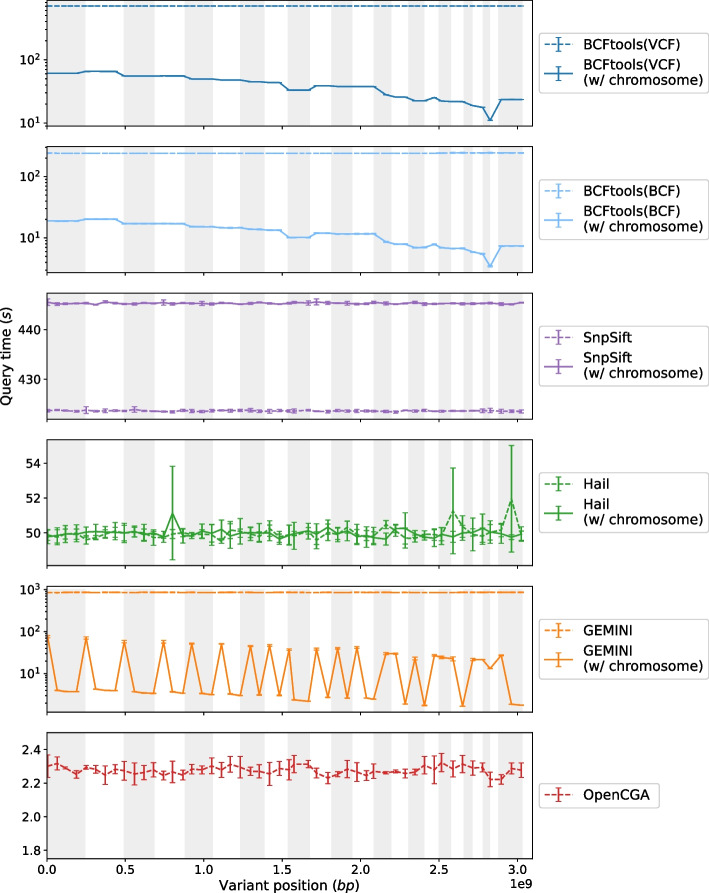
Query performance comparison for all studied tools to query for a unique variant by its identifier with and without providing the chromosome. Chromosome regions are shown as bands of dark and light rectangles. BCFtools and GEMINI results are presented in a log scale: as the query time between chromosome-bound queries and regular queries differ by order of magnitude, the log scale is more favorable to display the intricate patterns when querying with region indexing

**Fig. 3:**
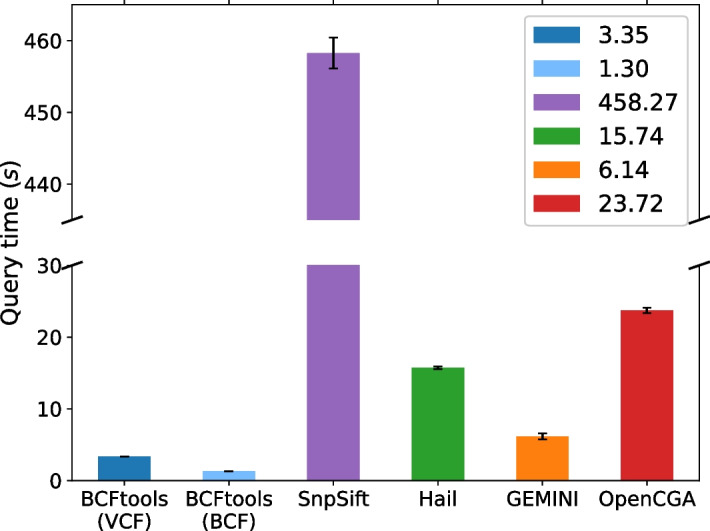
Query performance comparison between all studied tools to query for all INDEL-typed variants located in chromosome 5

**Fig. 4:**
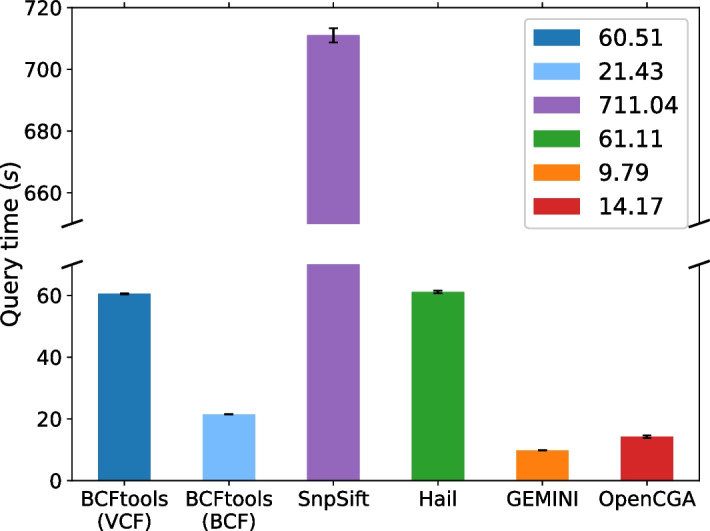
Query performance comparison between all studied tools to query for all variant sites where all samples in the study have homozygous genotype

**Fig. 5:**
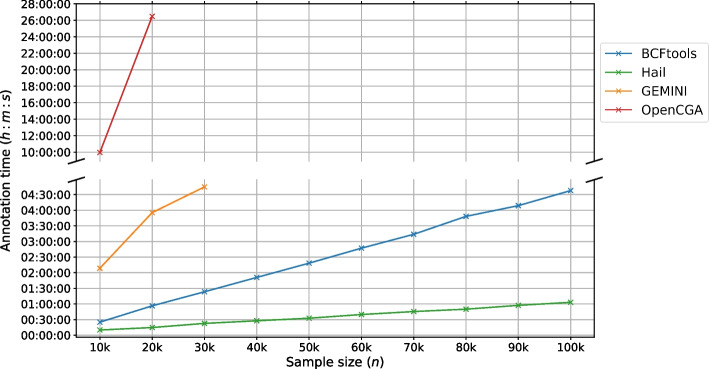
Time (in hours) taken by the studied tools to annotate the variants by patients and controls’ allele frequency. The annotation time is shown for a different number of samples

**Fig. 6:**
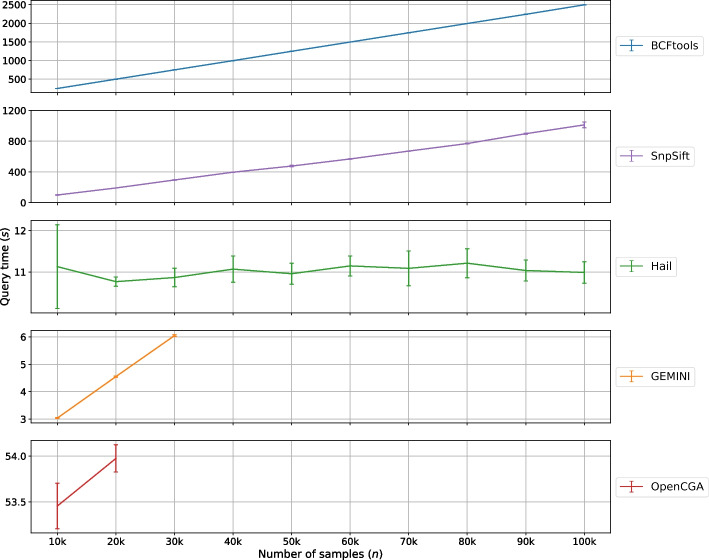
Query performance comparison of studied tools for different numbers of samples to retrieve all variants that appear in more than 40% of control samples and less than or equal to 40% of patient samples

A typical pipeline starts with a raw VCF after variant calling and consists of a sequence of four components: input, storage, annotation, and query. The input is the first step as the user provides the genomics data. In this context, it’s a VCF file in a human-readable format (uncompressed) or a binary/zipped memory-efficient format (compressed). The VCF file is then introduced to the tools to either be indexed or loaded into some form of storage. Some tools build upon the storage method by transferring the data from the VCF file into a designated data structure.

After the data is properly stored, the next stage is to augment the data with annotations (further explained in depth in “Annotation availability” section). The ultimate outcome following these steps is the ability to perform queries on variants and/or samples that take all sorts of associated information such as phenotype but also pathway membership between variants in the dataset. An example query would be to retrieve a set of variants based on the associated phenotypes and the sample genotypes. Other queries could be to find rare variants in a cohort, to perform a genome-wide association study, or PCA visualization of a cohort labeled by an annotation property.

### VCF data format

The VCF is a file format used for storing information about variants which are called with respect to a reference. As the human genome is known to be highly conserved from one person to another, the VCF file has been developed to represent the different positions in the genome where nucleotides vary. This format specification reduces the number of redundant nucleotides that would otherwise be identical in many samples. A properly constructed VCF file contains a header block (lines starting with the ‘#’ character), metadata arranged into columns (CHROM, POS, ID, REF, ALT, QUAL, FILTER, INFO, FORMAT), and one or more sample columns.

Each line in the metadata block details a variant that was called based on the alignments of sequences against a reference. The INFO column has an unstructured format that often contains reserved sub-field key-value pairs but is not strictly imposed. Users may define their own sub-fields as long as it is delimited by a semicolon and is defined in the header. The FORMAT column defines the fields for describing the samples such as the genotype, the read depth, or haplotype quality, and is uniquely determined for each sample in the VCF file.

GVCF (Genomic VCF) is another file format used by several variant callers, including GATK. It is a modified version of the VCF format that contains more comprehensive information about genomic sites, including non-variable sites. Further, gVCF files can be helpful to disambiguate situations where a variant is not called, either because the base calls agree with the reference or there was not enough (unambiguous) high quality coverage to make a call. Although gVCF files provide more extensive genomic information, they usually require conversion to VCF files before annotation and querying. The format is rather large and therefore often used only as a temporary file (e.g. during joint genotype calling in GATK). The additional storage, gVCF requires is another reason to have horizontal scaling designed into Genomic Data Science platforms.

### Annotation

Annotations are additional information that can be embedded into a VCF file. This enrichment of the VCF file is optional but crucial to the description of variants that are present in a call. Examples of such information pertain to the type of mutation, its associated phenotype, and cohort-related statistics (e.g. allele frequency in different world populations).

Annotations may be sourced in different ways: a common practice is to use publicly available annotation sources and copy over the information into the working VCF file. Annotation sources such as dbSNP [32] or gnomAD [33] contain annotations for variants that were previously explored for the association with a particular phenotype. Annotation transfer from such sources proposes the advantages of predicting whether a person is likely to have developed the phenotype. Mutation types—such as insertions, deletions, or single nucleotide polymorphisms (SNP)—can be inferred from the VCF file’s REF and ALT columns or from imported annotations [14]. These determine if the variant would have an impact on the overall expression of a protein. Another annotation approach is to calculate the allele statistics based on the sample genotypes. Sample statistics can also be captured from other cohorts and imputed into the VCF file from sources such as the 1000 Genomes project [34] (for cohorts originating in East Asia, North America, etc.)

### Tools

#### BCFtools

BCFtools (https://github.com/samtools/bcftools) was developed in 2009 as part of a consortium of related tools such as SAMtools and HTSlib. BCFtools is written in C and has amassed a large community of users due to its early adoption when bioinformatics tools were scarce. It is operated as a command line interface (CLI) tool and works directly on the VCF file without any transformations. Functionalities that target region-specific queries require that the VCF file be block-compressed and indexed. The indexing is then stored in the filesystem as a separate CSI or TBI format file. Variants are indexed solely by genomic position or isolate variants by scanning the entire VCF file for desired values in the INFO field. In addition, users can opt for the conversion of VCF to BCF file before processing.

#### SnpSift

SnpSift (https://github.com/pcingola/SnpSift) is a toolbox developed over 8 years ago by Pablo Cingolani. The tool is designed for extracting genomics data via VCF file manipulation and filtering. It is mainly written in Java, and the provided functionalities are aggregated in a Java Archive (JAR) file format and are run as a CLI program that operates on the VCF file directly. The community of users is fairly large which allows new users to familiarize themselves with the tool’s structure and syntax. Indexing variants and alternative storage options are not provisioned with this tool. SnpSift was also introduced as a continuation project to the annotation tool SnpEff, as a technique to query and filter the variant annotation information. SnpEff is a variant annotation tool that provides additional predictions for the effects of variants on genes. The idea was to create a two-step pipeline for users by using SnpEff to enrich the VCF file with annotations and then operate on the annotated file with SnpSift.

#### Hail

Hail (https://github.com/hail-is/hail) is a newcomer (relative to the other tools presented here) to the VCF manipulation scene and is maintained by a team of people in the Neale lab at the Broad Institute. The first cited use of Hail was in 2016 and it has been gaining traction over the years.

Hail presents itself as a Python library and employs the Matrix Table data structure for handling the VCF file data. Querying the data is akin to manipulating a Pandas DataFrame: using logical operations and string pattern matching as a means of filtering. The Python library can be installed via the Python package manager or built from a source which is necessary for some deployments.

#### GEMINI

GEMINI (GEnome MINIng) (https://github.com/arq5x/gemini) is a relatively modern framework designed in the Quinlan laboratory at the University of Virginia for human genome variations analysis and interpretations. GEMINI follows the workflow of a genomics data science approach like the one shown in Fig. 1. The VCF file is loaded into an integrated database where an automated process takes place by iterating over all the genetic variations and filling in the annotations for each variant. GEMINI utilizes a variety of annotation resources, which are listed in “Annotation availability” section. The tool employs an SQLite database to store variants’ information by mapping each field in the VCF file to a column in the database along with the newly added annotations. The interaction with the database is achieved through the “gemini query” tool where a basic SQL-like syntax can be written to construct the final query.

#### OpenCGA

OpenCGA (https://github.com/opencb/opencga) is the newest member of the genomic data science tools studied in this work. Developed by the Computational Biology Lab at the University of Cambridge, the big data platform aspires to solve major issues in scalability and performance with respect to genomics. Despite being a new player in this domain, the technology is already powering high-profile projects such as the 100,000 Genomes Project and is being used at Genomics England. OpenCGA is built in conjunction with a suite of tools known as OpenCB and includes a variant visualization tool (IVA) and an annotation server (CellBase).

In contrast to the other tools presented in this work, OpenCGA took the form of a solution platform. Genomic data is loaded into a management hierarchy of user-owned projects with multiple studies within a project; this enables access control to multiple users of the platform. OpenCGA leverages a networked object database to store all facets of information including sample identifiers, sample metadata, and variant information. The loaded data is then thoroughly indexed to enable fast querying and retrieval.

OpenCGA is supplied as a RESTful web service with multiple client options in Python, R, Java, and JavaScript. Queries can be done through the REST API using the language clients but also directly through the provided CLI.

#### Installation

BCFtools version 1.13 (https://github.com/samtools/bcftools/releases/tag/1.13) was installed into the working environment by downloading the release tarball from GitHub and compiled using Linux make tools. Likewise, SnpSift (https://snpeff.blob.core.windows.net/versions/snpEff_latest_core.zip) was downloaded as part of the SnpEff release zip file. Although no installation was required of SnpSift, Java is required to be installed in the system to run the JAR file. The version of Java installed was 1.8.0_222-b10.

GEMINI and Hail provide overall good documentation for the usage of the tools. Specifically, both provide a comprehensive set of examples for defining the syntax parameters. GEMINI is a standalone tool and Hail can be installed through the Python package manager.

GEMINI required an outdated version of Python (specifically 2.7.15) and a normalized VCF file. Python was used to run the GEMINI install script (https://gemini.readthedocs.io/en/latest/#new-installation) which was downloaded from the GEMINI website. Post-installation, the VCF file is indexed using Tabix [35] and loaded into an SQLite database using GEMINI’s CLI. As GEMINI has constraints to be installed on Python version 2.7, it is not likely able to stand the test of time as other libraries might deprecate functionalities that GEMINI depends on.

Hail (https://pypi.org/project/hail/0.2.105/) was installed using the Python package manager on Python 3.7.12. The raw VCF file was converted into a Matrix Table using a Python script featuring the Hail library functions. The Matrix Table is then saved onto the disk once converted for querying later.

OpenCGA is a relatively new tool; therefore the community of users is small and the documentation is limited to those found on the official documentation page. Furthermore, the tool can be installed in different configurations and could have many dependencies, increasing setup complexity. OpenCGA’s querying syntax is highly expressive but comes with a relatively steep learning curve. The tool’s initial challenge is compensated by the extra functionalities exclusive to OpenCGA. It is also well-supported for cloud deployment on Amazon Web Services and Microsoft Azure.

For our research setup, we endeavored to place each tool in a similar environment. To that end, we installed OpenCGA and its dependencies in separate virtual machines (VM): (1) a VM for MongoDB, (2) a VM for Solr as a secondary index, and (3) a VM for the OpenCGA tool in a Docker container. The version of OpenCGA installed was 2.1.0 (https://hub.docker.com/r/opencb/opencga-base), MongoDB is version 4.2, and Solr is version 8.6.0. The raw VCF file was loaded and indexed into OpenCGA using the CLI in the OpenCGA VM.

### Feature measures for comparison

A wide range of feature measures is covered to characterize various data science approaches. This in return brings a solid foundation for bioinformaticians and computer researchers interested in genomic analysis to identify different approaches that match their purposes. The feature measures with a brief description are listed below:

- *Scalability* This is one of the main standards that need to be considered highly in current genomic applications. In view of the fact that genomic data is consistently scaling, a compatible processing pipeline is necessary [9]. With this measure, we evaluated the scaling ability, and the type of scalability if applicable.
- *Data management* Here we identified the data storage options provided by each tool. We also distinguished between VCF flat-file-based, indexed files and database-based approaches and evaluated each option.
- *Storage of the INFO column* As mentioned in “VCF data format” section, the INFO column consists of numerous fields that describe the genetic variation in each row. Each field adds a different value to the overall understanding of a variant; hence, proper storage of the INFO column is desirable.
- *Data retrieval*
  - *Expressive power of queries* All the tools perform queries on information stored in a VCF file. We focused on the expressive power of queries; particularly, on queries related to the annotation in the INFO column (e.g. queries based on metabolism, type of mutation, clinical relevance, allele frequency, homozygosity, etc.). Those types of queries are considered complex as they are extracted using several fields in the database or the VCF file. In addition to these “INFO-column queries”, another kind of complex query are those involving secondary knowledge, for example: *extract all variants for samples belonging to a cohort, which are associated with Cardiovascular Diseases (i.e. spanning more than one disease identifier related to cardiovascular diseases)*. A simple query on the other hand is a query that extracts information from the common fields of a VCF file, targeting a single field and only referring to a single value. For instance, a simple query is to (*e.g. find all variants with allele frequency* $> 5\%$), or, (*e.g. retrieve all variants with heterozygous genotype for all samples*).
  - *Query speed* This is another measure that is considered essential and most often overlooked in comparison to annotation speed. Complex queries, such as querying for variants of polygenic diseases, can vary in response times due to varying indexing schemes. Measuring the query throughput will shed light on the efficiency of the indexing deployed by the tool.
- *Entry requirement and installation* This measure tests the availability of documentation to aid in setting up and using the tool. Furthermore, it identifies the infrastructural requirements to get the tool operational. It also determines the tool’s learning curve and the complexity of navigating through the tool’s functionality.
- *Annotation availability* Typically, variant annotation is part of the process of genotype data enrichment. Consequently, some VCFs have annotation information already present in the file. In this measure, we were particularly interested to show if the tools make use of the internal annotation in the file. Additionally, we indicated if the tool is capable of integrating external annotation sources. The relevance and the size of the sources are also evaluated.
- *Customization (functionality and database)* In addition to the measures defined above, we tested if the studied tools provide room for functionality and database extension. It is common to tailor a pipeline to meet different objectives after acquiring a VCF file. Several applications claim to provide a customizable analysis platform, and this feature metric verifies that.
- *Output* The output content is formatted differently in each genomic application. We used this feature measure to inspect the readability, usability, and completeness of data portrayed in the different output formats.

## Results

In this section, we explore the data preparation and system specifications to set up each tool. Furthermore, we provide a detailed evaluation for each of the feature measures mentioned in “Feature measures for comparison” section. All the findings are reflected in a detailed feature comparison Table 1 while considering the general analysis pipeline shown in “General workflow” section.

### Data preparation and system specification

An in-house bioinformatics unit was built to develop a Genome Sequencing analysis pipeline on High-Performance Computing and large servers. More details on the data can be found in an article by Daw Elbait et al. [36]. Data sourced from the output sequencing of machines in the bioinformatics unit (e.g., Illumina NovaSeq, NextSeq, and MiSeq) is streamlined securely into a variant calling pipeline that features industry-standard tools.

The testament to this pipeline is a curated set of sequences from 153 (120 genomes and 33 exoms) UAE nationals. This data, consisting of over 25 million variants, was used as a standardized data input to analyze and compare the genomics data science approaches in this work. For one query scenario, we leveraged a data array of 805,426 variants sourced from the UK Biobank (UKB) [37]. This enabled us to evaluate the tools’ capabilities on a large scale in the dimension of sample size. We selected patients and cohorts based on ICD codes. This captured roughly 100,000 patients and controls which became our primary dataset. Through random subsampling, we built smaller cohorts of patients and controls of sizes 10,000 up to 100,000 in increments of 10,000.

We validated the performance of five data science solutions, namely BCFtools, SnpSift, Hail, GEMINI, and OpenCGA. The constant across all tools is the initial VCF file that is processed and queried by each tool. Different tools may require further processing of the VCF file into either a copy or into another structural format containing the data in the original VCF. A summary of each tool is reported in “Tools” section. All solutions were installed on a local Linux server with the following system specifications: Intel Xeon Gold 6130 32-core CPU @ 2.10 GHz and 64 GB RAM, running CentOS 7. All parameters, including the input file, were consistent across all running tools.

### Evaluating feature measures

In Table 1, we showcase the findings of this comparative analysis. We have prepared and summarized the features of each tool according to a predefined set of feature measures (see “Feature measures for comparison” section). These measures described the tool’s performance and operation starting from the entry requirement phase, through to the downstream analysis. The purpose of this comparison is to create a benchmark for data science researchers to identify the optimal approach for their scope of work. It also works as a reference for designing new genomics data science applications. We took into consideration the overall flexibility of the analysis pipeline for each tool, the repeatability of the process steps, and the time it takes to execute various queries. In addition to the summary in Table 1, the following is a detailed description of the findings for each evaluation measure.

**Table 1:** A summary matrix of the tools presented in this work along with the different feature measures on which the tools are evaluated

	OpenCGA	GEMINI	Hail	Bcftools	SnpSift
Entry requirement and installation	Many dependencies	Python-based install script	Python package installation	make compilation	Java JAR download
Data management	MongoDB/HBase	SQLite	Matrix Table	Flat-file VCF	Flat-file VCF
Storage of the INFO column	Highly indexed, nested object structure	Partially indexed SQLite tabling	Stored as type-inferred columns	Unindexed VCF file INFO column	Unindexed VCF file INFO column
Annotation availability	34 data sources, manual	18 data sources, automatic	13 data sources, manual (experimental)	N/A, manual	dbNSFP, manual
Query complexity	Multiple clients, unconventional syntax	SQL query-like	DataFrame-like filtering	CLI, documented syntax	CLI, documented syntax
Query speed	Fast, comprehensive indexing	Database indexing, moderate speed	Fast, Spark-backend querying	Fast for flat-file based query, indexes by chromosome	Slow, not indexed
Query ranking	Best in rsID query (Scenario 1)	Best in homozygous genotype query (Scenario 3)	Best in complex query (Scenario 4)	Best in INDEL-type query (Scenario 2)	Overall last place
Scalability	Horizontally scalable, managed platform	Limited vertical, monolithic	Efficient filesystem storage, Spark-based	N/A, monolithic	N/A, monolithic
Customization (function and DB)	Java plugins	Only DB is extensible	Python-native	C plugins	Only built-in commands
Output	JSON, VCF-like, Tabular text	Tabular text	Matrix Table object	VCF file, Tabular text	VCF file

#### Scalability

BCFtools and SnpSift are single programs that are built for manipulating flat VCF files. BCFtools additionally have the ability to manipulate flat BCF files. This means the limitations of these tools are realized as the data size grows by samples or variants. Another drawback is the extra efforts brought by the need to provide the annotation sources and then reintroduce them to the expansive VCF/BCF file. These tools naturally have no networking capabilities.

Hail employs the Matrix Table which stores a data structure that references the actual data. Operations on the Matrix Table are planned out but deferred until users request the output. Although it may not always be fast, it scales well when used with large datasets. Downstream processing can be distributed via a Spark local instance or Hadoop cluster; however, Hail lacks data distribution in terms of networked storage. For larger-scale projects, Hail will require much more time to construct the initial Matrix Table. Beyond that, Hail primarily relies on vertical scaling as larger projects require more storage space and more CPU capability to perform as expected.

GEMINI is competent in small-scale situations when the number of variants is low. GEMINI lacks networking capabilities due to the design choice of SQLite as a database management system, despite the availability of other database alternatives like MySQL, PostgreSQL, or Vertica [38]. This minimizes the amount of horizontal scaling as one can not provision more machines to increase throughput. Scalability is achieved by installing better CPU, memory, or storage as a means of storage expansion or processing queries more rapidly.

OpenCGA is highly scalable as the storage is built with horizontal scaling in mind. Storage is supported by distributed database services such as MongoDB and HBase, thus allowing processing pipelines to exploit the networked nature of the platform to perform tasks in parallel. Samples and variants are stored in the distributed database as documents containing the INFO column and other metadata. The modular design enables the further addition of samples and variants after data has already been loaded and established.

#### Data management

The data storage options provided by each tool are considerably diverse. BCFtools and SnpSift operate on the VCF flat file without any extract-transform-load (ETL) mechanism. Advanced uses of BCFtools require that the files be block gzipped which is doubly beneficial: it reduces overall disk storage and enables swift access to variant loci (assuming the VCF is sorted). BCFtools allows the user to transform the VCF file into BCF format which trades off a slight increase in disk space for faster processing. BCFtools and SnpSift rely on indexing, but the indices are stored in the filesystem. Operating directly on the VCF file will affect the speed as is discussed in “Query speed” section.

The rest of the tools follow an ETL procedure. Hail utilizes the Matrix Table; a distributed data structure that resembles matrices and Pandas DataFrames [18]. The original VCF file is transformed into a multidimensional Matrix Table which is saved as a set of files containing schema description in JavaScript Object Notation (JSON) and raw data in binary format.

GEMINI loads and stores the VCF file along with the variants’ annotations in an SQLite database. The database consists of seven tables, with the main one being the “Variant” table. The database schema is provided in the documentation available on the tool’s website. GEMINI stores genotype information as a binary blob especially for info that relates variants to samples. The variant table is indexed based on the locus, combining the chromosome number and position.

OpenCGA manages the VCF data and stores it in an object database. Sample information and variant information are stored in different collections in the OpenCGA “Catalog”. There are two usable storage setups (i.e. MongoDB [39] and HBase [40]). Object databases store nested key-value pairs, allowing for unstructured information to be stored efficiently. OpenCGA creates a unique identifier for variants and distinguishes between multi-allelic variants by creating a new record for each allele. An example of a variant identifier format is as follows: <chromosome>:<position>:<reference allele>:<one alternative allele>

Applying these tools to our local genomic data composed of 150 Emirati genomes, we recorded the total storage utilization in Table 2.

**Table 2:** Storage utilization of each tool after transformation

	Storage use (GB)	Annotations	Notes
Original VCF	93.77	No	–
BCFtools(VCF)	16.75	No	bgzip + csi file
BCFtools(BCF)	19.60	No	bgzip + csi file
SnpSift	16.75	No	Same as BCFtools(VCF)
GEMINI	119.48	Yes	SQLite file size
Hail	17.55	No	Matrix table folder size
OpenCGA	103.27	Yes	MongoDB collection size

#### Storage of the INFO column

A common annotation practice following the GATK Best Practice Pipeline applies several annotation tools sequentially. From an information theoretic perspective, this practice is disadvantageous. Not only does it leave a trail of large, unindexed, and highly redundant files, but it also often leads to inconsistent INFO column formatting due to conflicting conventions. As a result, this practice does not lend itself to fast querying.

Some examples of the abuse of INFO columns include the interchanging of separators, mixed word casing (snake case, camel case), and subfield annotations using period marks (e.g., mydb.annotation=...). Additionally, this information is stored as text as opposed to industry-grade data structures that accelerate querying retrieval. Therefore, it is preferable to normalize the extract-transform-load (ETL) process that standardizes information entry, especially with respect to the INFO column when the data scales up.

BCFtools and SnpSift do not have special indexing for the INFO column, rather they operate directly on the INFO column in the flat VCF file or BCF file in the case of BCFtools. However, they also allow users to query the INFO sub-fields in a convenient way through built-in subcommands. The query mechanics will be explained in a later section.

Hail stores the INFO column into typed columns as part of the Matrix Table. The INFO columns are identified based on the header of the loaded VCF file and the data type of the INFO sub-field is automatically inferred based on the available data. The INFO sub-fields are accessible through the Matrix Table loaded into memory when running in a Python environment.

GEMINI stores the INFO data under designated SQL table columns built into GEMINI. For the remainder of sub-fields in the INFO columns that do not match the GEMINI predefined table columns, a BLOB field is provided as general storage.

The INFO column is stored in OpenCGA as a collection of objects in the Catalog. Each INFO sub-field is stored takes up space in the flexible data schema, and inherits the exact formats as specified in the meta-information part of the VCF file.

#### Expressive power of queries

We recalled the query examples stated in “Feature measures for comparison” section in order to verify if each tool can execute various complex queries.

With BCFtools, users can build complex queries provided that the querying VCF/BCF file has sufficient annotation and adheres to the assumed format conventions. BCFtools allow for numerical value filtering, and regular expression matching for multi-faceted INFO subfields and can combine queries through logical operators. Note that the multi-faceted INFO subfields are unindexed and therefore depend on the correctness of the regular expression. This is error-prone as similar fields such as AF and MAF can match the same query giving false positive results.

SnpSift does not facilitate the use of complex queries. The tool is more focused on VCF manipulation, such as calculating the concordance between two VCF files, joining two VCF files, the intersection of variants sets in VCF files, and so forth.

The Matrix Table designed by Hail is structured in a usable way for building complex queries. Users are able to list all values of the INFO sub-fields in tabular format and inspect each INFO sub-field. Operations typical of a DataFrame can be conducted on the Matrix Table to filter, sort, and aggregate data across the VCF.

With GEMINI, the complexity of the query is bounded by the limitations of SQL queries and the capacity of an SQLite database. Generally, SQL has an overall high expressive power and for the purposes of querying information in a VCF file, SQLite is sufficient to perform complex queries.

OpenCGA queries are executed on the object database storage of variants with multiple techniques provided. The primary mode of querying is via the CLI, although alternative modes of querying are available through a REST API call, and Python or R wrapper library. Note that all the above-mentioned query mechanisms can be combined with GATK workflows, though with varying efforts: BCFtools and SnpSift operate on (position-indexed) VCF files directly, whereas the latter three approaches require database population of GATK’s VCF files.

#### Query speed

We tested the query speed by running several scenarios using the same dataset across all tools. To make a fair comparison, we used the Linux time command to measure the response time of each query starting from user input to output. This includes the total amount of time for the tool to load the variant data from its respective storage type, execute the query and return the result. We recognize that some tools have a longer startup time but could return a query more quickly if the data was already loaded into memory (particularly for Hail).

In some cases, BCFtools query time was consistent and performed well in comparison to other tools due to optimizations in the implementation written in the C programming language, and chromosome region indexing. However, BCFtools operates line-by-line and still needs to go through the entire VCF/BCF to produce an output. The query time is notably reduced when an index is provided (e.g. chromosome region) since BCFtools has to go through the entire chromosome region instead of the whole VCF/BCF file. It was also notable that the query time would fluctuate proportionally to the chromosome region size. It is also notable that BCFtools queries faster with BCF files. However, the conversion time from VCF to BCF is about one hour for our dataset. Additionally, queries that are accelerated by region indexing require that the VCF file be compressed using bgzip and indexed.

SnpSift does not perform automated indexing; hence, the query speed is the slowest among the tools. For instance, that can be seen when two identical queries are executed on different chromosome regions. For example, a query that targets variants in chromosome 1 takes much less time to display on the screen compared to a query targeting chromosome 13. This is because the VCF file is ordered by the chromosome loci in an ascending manner. However, in both cases, SnpSift would take similar amounts of time before stopping its execution (as it had to complete the entire VCF file). This is normally remedied by splitting the original VCF file into smaller VCF files consisting of each chromosome region but increases the difficulty in data management.

It is noted that Hail’s indexing is considerably better than GEMINI’s. We speculate that Hail fully exploits its multi-threaded workload via Spark while GEMINI suffers from a lack of performance as the indexing is done on an SQLite DB which is not fully managed like MySQL or PostgreSQL. Another point to be addressed is that all Hail query speed results are inclusive of the initialization time of 6 s.

OpenCGA query speed is considered the highest among these tools on average. This is attributed to OpenCGA’s feature of indexing multiple fields when a VCF is loaded and annotated. It should be noted that OpenCGA’s query via the command line performs a REST API call which can introduce delays in the overall execution time.

Figures 2, 3, and 4 illustrate the query speed for Scenarios 1, 2, and 3 respectively.

**(a) Scenario 1**

*Return all information for a variant given its unique rsID*

We evaluated the five tools to query for a unique rsID throughout the whole genome. The query was performed in two different manners: including the chromosome region as a query parameter, and by querying solely with the rsID. As some tools index by chromosome regions, it would be interesting to identify what performance gains can be realized by leveraging the indexing. We randomly sampled 50 rsIDs across the entire genome that are roughly equidistant. These rsIDs were sampled along with and without their respective chromosome region. Queries were run in the order that the selected variants appear in the genome and each query is repeated 5 times to measure its statistics.

The general trend across all tools is that queries involving the chromosome region of the targeted rsID are faster than those without. The exception to this is SnpSift whereby the query with the chromosome region is about 20 s slower than the query without the chromosome region. We assume that this is due to the complexity introduced into the query to not only check for the right rsID but also to check that the chromosome matches correctly.

It is evident in the first plot of Fig. 2 that BCFtools employs indexing when the chromosome region is present in a query. Within a chromosome region, query run times plateau, suggesting that BCFtools explores the extent of the entire chromosome for each query. Naturally, smaller regions are much quicker to query compared to larger regions. Note that the size of the chromosome is not indicative of the query speed but rather the number of variants that were called in that region. As shown in Fig. 2, querying VCF and BCF files result in a similar trend across the chromosomes. However, when it comes to query speed, BCF files outperform VCF files.

The difference in delay between the chromosome-bound query and regular query is noticeable across all tools except for Hail where the running times for each query are almost identical. Peaks can be seen at different positions across the genome but these values fall within the bounds of the standard deviation of the individual runs. This is possibly due to background processes occupying the CPU when the query is run.

Without the chromosome region available, GEMINI appears to scan through the entire SQLite database for the matching rsID, akin to BCFtools and SnpSift. However, an interesting fluctuation pattern appeared as queries were executed across the genome when the chromosome region is present. It is noticeable that these fluctuations appear to be highest at the start of a new chromosome. That might be due to the way GEMINI stores information between queries. Queries are seemingly dependent on the last information kept in the cache. As long as the next targeted rsID belongs to the same chromosome as the previous query, the variant can be returned quickly. However, as noticed in Fig. 2, when the chromosomes changes, GEMINI requires time to load the new region into the cache and thus slowing down the query speed for the first retrieved rsID in the next chromosome.

OpenCGA is the fastest among all tools in this plot. The tool maintains its speed results in both types of queries (with and without chromosomes) under a total of 2.5 s and there is a sustained 2-s delay between the two types of queries. OpenCGA retrieves the variants consistently across the whole genome as is evident by the straight line performance in Fig. 2.

**(b) Scenario 2**

*Get all variants typed INDEL in chromosome 5*

With this test, we evaluated the performance of retrieving indel-typed variants given that the original VCF did not contain that information. Querying for variants by their variant type (e.g. SNP, SV, INDEL, etc.) is a common analysis to identify potentially harmful mutations when proteins are translated from the RNA sequence. We have arbitrarily chosen chromosome 5 to limit the search space of the query. This query is repeated 5 times to account for variability between runs.

Overall, BCFtools is the fastest despite the lack of well-defined variant type information in the INFO column. As the tool streams over the VCF/BCF file, it determines the variant type by comparing the REF and ALT columns. This query is enabled by the powerful “expressions” functionality featured in the BCFtools documentation, which would have otherwise been unknown. BCFtools is likely fast due to its implementation in the C language and runtime optimizations. Again, we see that the BCF file responds faster to this query, compared to the VCF file.

SnpSift lacks the functionality to immediately compare the REF and ALT columns. Instead, one would need to annotate the VCF file using the VarType command to populate the variant type field in the INFO column. This step yields an annotated VCF file that is different from the original. This annotated VCF file is then queried for the variant type to find all variants which are INDEL. The time displayed in Fig. 3 does not take into account the time to generate the annotated VCF file.

Hail provides a built-in function called is_indel to determine if the reference and alternative alleles are indels. This function is applied to the “alleles” column of the Matrix Table and is used to create a boolean value column that indicates whether a variant is an indel or not. The query is then executed on this new column to filter for indel-typed variant rows. Hail’s query time also includes the time taken to create the boolean column and the time query to query by that column; hence why it is slightly slower than BCFtools and GEMINI.

GEMINI’s database contains variant type information as a result of the initial annotation when loading a VCF file. The query is executed against the “type” column in the “variants” table and the run time is not capturing the annotation step. GEMINI’s use of SQL tables is of notable value in this situation where there are few possible values for a single column.

Like GEMINI, OpenCGA creates the variant type as part of the initial annotation process. Although OpenCGA has extensively indexed the VCF information and annotation, the query executed against the variant type field is surprisingly slower than BCFtools which operates on a flat file. Additional space requirements to store the variant type for each tool are shown in Table 3.

**Table 3:** Additional space requirements to store the variant type for each tool

	Additional space requirement	Rating
BCFtools	None	Low
SnpSift	Creates a new VCF file with INDEL boolean flag	High
GEMINI	None, pre-annotated during initial load	Low
Hail	Creates a new column of boolean type in Matrix Table	Med
OpenCGA	None, pre-annotated during initial load	Low

**(c) Scenario 3**

*Retrieve sites where all samples have the homozygous genotype*

In this scenario, the query checks that all samples have the genotype 0/0 or 1/1. This tests the tools’ capability to handle queries spanning multiple, if not all, samples in a VCF file. This particular query is thought to be useful for determining if a cohort collectively has a different genetic makeup compared to the reference and is useful for classifying groups. To minimize variance in the results, the query is run 5 times.

BCFtools—querying a VCF file—and Hail demonstrate similar performance, with Hail having a 6-s advantage over BCFtools due to its start-up delay. However, when working with the corresponding BCF file, BCFtools exhibits faster processing compared to Hail. SnpSift appears to have its worst performance over all queries studied in this benchmark.

GEMINI utilizes a Python indexing package called bcolz to speed up queries targeting genotype fields. bcolz is based on columnar, compressible, chunked data containers. Genotype columns in the GEMINI database are indexed to accelerate querying using the argument “–use-bcolz” in the same genotype filtering query to get a quick query response. That is shown in Fig. 4 as GEMINI scores the highest speed among all tools. Skipping the indexing step, however, would increase GEMINI’s query time dramatically up to an average of 150 s.

OpenCGA is second to GEMINI in this test scenario. However, it is a considerably slight difference between the two tools.

**(d) Scenario 4**

*Retrieve the variants where the allele frequency of patients is below or equal to 40% and the allele frequency of controls is above 40%*

*(i) Annotation time*

In this scenario, prior to the query, a calculation is required to annotate each cohort (patient and control) by the allele frequency. The query then retrieves variants based on the annotation which is found in the INFO column of a VCF file. This tests the tools’ capability to handle queries spanning multiple, if not all, samples in a VCF file. The number of variants remained constant throughout all sample sizes. This particular query is thought to be helpful in determining if a cohort collectively has a different genetic makeup compared to the reference and is useful for classifying groups.

From a biological perspective, this query identifies variants that are not likely to appear in patients of a study. In our case, we investigated patients with circulatory system disorders in the UK Biobank dataset. We tested the tools’ capabilities in terms of two aspects: the annotation time and the query time as shown in Figs. 5 and 6, respectively.

Hail is the fastest among studied tools in terms of the time taken to process the annotation and save it into the Matrix Table format. BCFtools comes second, and the annotation is simply inserted into the INFO column of the VCF file. Both Hail and BCFtools have a linearly correlated annotation time which increases as the sample size grows.

SnpSift does not provide any command to annotate the variants based on the cohort’s allele frequency. However, we reused the annotations provided by BCFtools to measure the query time for SnpSift. As for GEMINI, we performed annotation for sample sizes 10k, 20k, and 30k and did not proceed further due to the prolonged annotation times. It took more than 2 h for 10k samples to be annotated and almost 5 h for 30k samples. This annotation time combines the time taken to load the VCF file to an SQLite database, and the time it takes to update the database by inserting new columns for the control and patient allele frequencies.

OpenCGA had the slowest annotation time due to the several steps involved to annotate the cohorts. The steps include linking the VCF file, the primary indexing of variants, cohort creation, and statistical calculations to generate the cohorts’ allele frequency. We also had to limit the annotation process for OpenCGA up to a 20k sample size. One reason is that the time taken to process a VCF file with 20k samples requires more than 24 h to complete. Another reason is the tremendous amount of RAM that is required to run the indexing which is in excess of 32 GB. The true value of the memory that would be required is unknown as we did not have the capacity to provision more memory to the virtual machine.

*(ii) Query time*

Across the 5 repeated runs, all tools exhibit a linear increase in query time, except Hail, with respect to the number of samples. BCFtools query time ranged between 8 min up to 42 min across all sample sizes. SnpSift performed surprisingly well in comparison to its performance in all other scenarios presented in this work. The query time increased from 2 min to under 20 min between 10k to 100k samples. As the content of the VCF file increases between 10k samples and 100k samples, the number of bytes that needed to be processed by SnpSift and BCFtools increases; therefore leading to an increase in query time. This is one of the inherent weaknesses of using flat-file (e.g., VCF) storage for querying data in the INFO column.

Hail is considerably fast among the studied tools and is consistent across all sample sizes. We attribute this to Hail’s ability to store allele frequencies as floating-point data in the Matrix Table once it has been annotated. Hail leverages Spark in the backend to parallelize queries and enable faster retrieval across all chromosomes. As all datasets contained an identical number of variants, the small fluctuations in query time are visible artifacts of random CPU throughput during different runs.

The query time for GEMINI is measured for the previously annotated 10k, 20k, and 30k sample size databases. The highest query time goes up to 6 s for the 30k sample size database which is considered very fast in comparison to the other tools. The way SQLite database stores floating-point data appears to have some benefits for quick retrieval of data.

OpenCGA query time results for 10k and 20k sample sizes seem consistent, though there is too little data to extrapolate further whether it would be linearly correlated with sample size or not. It is worth mentioning that OpenCGA introduced a timeout error when the query was set to retrieve all variants. To tackle this, we had to limit the number of variants in the query in order to include OpenCGA in this analysis. The same limit of the first 100 variants encountered was set for the queries in all other tools to ensure a fair comparison.

#### Entry requirements and installation complexity

In this feature measure, we mainly evaluated the availability of documentation for setting up and using the tool. We found BCFtools and SnpSift to be the best among the five tools in providing detailed documentation for users. These two tools benefit from having a larger community of users as a result of being developed prior to the others. New users trying to get familiar with the syntax can easily find solutions to their problems through numerous results in search engines. In addition, these tools are standalone, command-line interface (CLI) based programs and have few dependencies. Installation complexity as described in “Installation” section is summarized in row 1 of Table 1.

#### Annotation availability

BCFtools have the functionality for annotating a VCF/BCF file but do not adhere to any specific annotation source. SnpSift is coupled with dbNSFP [41] and provides a subcommand to annotate VCF files with the rich functional predictions found in dbNSFP. For both tools, users may employ any desired additional annotation source, with the condition that the source is formatted to the tools’ annotation format specifications.

Hail features a moderate set of annotation sources coupled with the library, but the functionality is currently in the experimental stage. The annotations are hosted online in the cloud and users must have a Google Cloud Platform (GCP) or Amazon Web Services (AWS) account to operate the annotation database class. Annotations are applied to the Matrix Table and the information is saved into the filesystem as described earlier in “Data management” section.

GEMINI boasts a large set of annotation sources from notable public annotation archives such as ENCODE [42], UCSC [43], OMIM [44], dbSNP [32], KEGG [26], and HPRD [45]. Upon loading a VCF file, GEMINI automatically annotates the variants and stores the result in the internal SQLite database. However, GEMINI only supports human genome variants aligned to build 37 (hg19).

OpenCGA draws annotations from a comprehensive library via CellBase [46]: a NoSQL-based annotation database that compiles numerous annotation sources comparable to the popular Ensembl Variant Effect Predictor (VEP) tool [22]. Users must configure the CellBase server connection credentials if the users have a local CellBase installation. Otherwise, annotations are collected from a publicly available CellBase server hosted by OpenCB but the process risks delays due to connection latency. The set of common variant annotation sources present across each tool is listed in Table 4.

**Table 4:** Common annotation sources available in each tool

	OpenCGA	GEMINI	Hail	BCFTools	SnpSift
1kG	$\checkmark$	$\checkmark$			
CADD	$\checkmark$	$\checkmark$	$\checkmark$		
ClinVar	$\checkmark$	$\checkmark$	$\checkmark$		
COSMIC	$\checkmark$	$\checkmark$			
dbSNP		$\checkmark$	$\checkmark$		
Ensembl	$\checkmark$	$\checkmark$	$\checkmark$		
ESP	$\checkmark$	$\checkmark$			
GERP	$\checkmark$	$\checkmark$	$\checkmark$		
gnomAD	$\checkmark$	$\checkmark$	$\checkmark$		
Polyphen	$\checkmark$	$\checkmark$			
Others	$\checkmark$	$\checkmark$	$\checkmark$	N/A	dbNFSP

#### Customization (function and database)

BCFtools allow for functionality customization via the development of plugins in the C programming language. An API template is provided to standardize the structure of custom libraries. Users can use the internal functionality of other SAMtools-related libraries such as HTSlib, SAMTools, and BCFtools. Currently, there are custom functionalities that are community-built and accepted into the BCFtools repository such as counts, indel-stats, and remove-overlaps to name a few.

Being a monolithic tool, SnpSift only provides built-in commands which are accessible to the users via the CLI. However, the developers have made a wealth of functionality available in SnpSift which users can leverage in scripts to compensate for the lack of customizability.

Since Hail is a Python-based data analysis library, there is the flexibility of using other libraries in conjunction with Hail. One example would be leveraging plotting and eager execution during the analysis of genomic data in the comfort of a Jupyter notebook environment. The Matrix Table can be modified to include user-defined columns which provide the freedom to customize the INFO column sub-fields programmatically.

For researchers, the main customizable functionality in GEMINI is given by the option to include custom annotations via sub-commands. The tool also shows flexibility in terms of allowing users to extend the SQLite database by adding columns to any GEMINI pre-defined table or creating new tables to suit users’ personalized analysis.

OpenCGA welcomes community-developed plugins as a way to extend the platform. Java plugins can be added to the internal processing pipeline, thus operations can be performed directly on the query iterators, increasing the efficiency of the novel functionality that benefits from this access. Moreover, the Python and R wrappers give flexibility to analysis options in the respective languages, although this would come at the cost of speed.

#### Output

BCFtools and SnpSift output the data in the same format as the input VCF file, in addition to tabular formats that are viewed with some query types. If BCFtools “view” is used, whole VCF/BCF files can be almost entirely regenerated creating redundancy in the storage as rows of the VCF/BCF are extracted.

Hail produces a structured tabular output format by default, where the nested INFO columns are also indicated. Users may also export the output as a Matrix Table or other genomics-specific file formats such as VCF, PLINK, and BGEN. Hail also provides some visualization functionalities that are featured through the Jupyter notebook environment making it an additional output presentation. For example, Matrix Tables can be rendered as HTML tables which suits the conventions of a Jupyter notebook.

GEMINI’s output format is a typical SQLite tabular text output. Users need to specify the column fields that would appear in the output or choose to view all columns. OpenCGA provides many output format options for users. The default output is a VCF-like structure though this option lacks some genomics information such as the annotation and the variant unique ID. As an alternative, users may choose between JSON format or tabular text format to view the detailed data of variants. It is important to highlight that OpenCGA holds all the information on gene-related traits but these annotations do not appear unless the user provides at least one of the traits in the query. For example, a user would provide at least one OMIM ID in the query in order to get all other related OMIM IDs.

## Discussion

We note that the feature measures mentioned in this work are not exhaustive and there could be many other facets that put a tool in a different light. After consideration of how the tools are deployed, we believe these metrics shed light on the typical questions posed in a modern Genomics Data Science project. We strived to deliver a fair comparison between all the tools to the best of our ability, though in certain situations we had to work within the constraints of the tools to achieve our results. Another promising tool, somewhat resembling the functionality of GEMINI, is slivar [17]. It has strong capabilities to query and filter variants and has a strong focus on rare diseases and facilitates analyses with filters provided by the hts-nim [47], a library accessing htslib. Slivar does not utilize state of the art database technology and doesn’t address population wide scalability and fast data retrieval, in particular, it does not focus on horizontal hardware scaling of genomic projects. As the tool requires a pedigree structure (and in particular, a pedigree file), slivar is not directly compatible with the structure of the population scale benchmark used here.

As for the future of large scale genomics projects, it is conceivable and—in our opinion—desirable that variant information including secondary annotations are stored, indexed and queried as database operations, thus leveraging the mature and scalable database technologies of recent decades. This process should start as early as variant calling in genome pipelines. For example, popular VCF generating tools like GATK can benefit from database operations during variant calibration, variant filtration, and annotation. The current best practices often involve recreation of new versions of VCF files that are largely redundant thus, not storage efficient.

Efforts to use database technology (GenomicsDB) have been made in GATK version 4.0, but are currently not used to its full potential. The principle then transcends to downstream steps such as genotype phasing and cohort selection, all of which can be couched as database operations. The modularity of a database platform with variant data and additional information in respective tables is also convenient in terms of progressive annotation extension and update: e.g., for relational databases, the discussed annotation sources (Table 4), which often are already available in proper database format (SQL, tabular formats), can be integrated with conventional and highly optimized join operations. In summary, we argue that it is beneficial for genome analysis platforms like GATK to be tighter integrated with Big Data technology.

## Conclusion

In this work, we evaluated the performance of several genomics data sciences approaches to interpret and extract variation data. We studied five tools, namely SnpSift, BCFtools, Hail, GEMINI, and OpenCGA, and compared them according to several feature measures to display the tools’ qualities in the context of Genomic Data Science. Among the aspects of a desired data science tool are the entry requirements, query-related features, storage paradigm, annotation capabilities, and scalability. The key feature is the expressive power captured across all tools to enable the transformation of a researcher’s genomics questions into explicit, yet efficient queries.

It is evident that preprocessing of the INFO column into appropriate data structures that leverage indexing enjoy numerous benefits. Among those seen in this work are: performant queries, better scalability, more flexible pipelines, increased operational integration, and enhanced protection against data mishandling. The trade-off to achieve these merits is primarily in boosting storage capacity which, in modern systems, is not excessively costly as many genomic data stores currently relish vast storage prescriptions [48].

It bears mentioning that the apparent Achilles’ heel for modern genomic querying tools is complexity. Complex systems incur great technical debts, thereby entailing more expertise and personnel to manage, hindering adoption. Tools like Hail strike a good balance between sophistication and usability by keeping a low technical footprint with the conveniences of structured data. At the two extremes are BCFtools: the locally-hosted monolith with a low learning curve, and OpenCGA which span multiple services but is highly query-optimized.

We found that different tools suit different situations and resources. Thus, users can opt for the appropriate tool depending on the scale of their projects. Our results show that flat-file manipulation tools are less preferable in comparison to tools that leverage sophisticated data organization in various aspects. The paradigm that OpenCGA has adopted supports horizontal scalability in large-scale projects like the 100,000 Genomes Project [49] and UK BioBank database [37]. However, current implementations leave a large room for improvement in terms of usability and community strength.

## Data Availability

Data supporting the conclusions of this article is available used here was published at the European Genome Archive (EGA) under study accession number EGAS00001004537 with the dataset link: https://ega-archive.org/datasets/EGAD00001010916. Genotype datasets from UK Biobank were retrieved under application number 64823. The data is subject to UK Biobank regulations.

## Code availability

Benchmark scripts are available at https://github.com/syaffers/genomic_ds_queries. The github repository also contains a docker file that containerizes all benchmark scripts and tools (except OpenCGA which requires additional setup).
